# Feasibility Test of a Customizable Relationship Intervention for Stroke Survivor-Family Caregiver Dyads

**DOI:** 10.1093/geroni/igaa057.1205

**Published:** 2020-12-16

**Authors:** Michael McCarthy, Yolanda Evie Garcia, Karen Lyons, Angelica Sanchez, Tamilyn Bakas

**Affiliations:** 1 Northern Arizona University, Flagstaff, Arizona, United States; 2 Boston College, Chestnut Hill, Massachusetts, United States; 3 University of Cincinati, Cincinnati, Ohio, United States

## Abstract

A strong interpersonal relationship after stroke is important for the well-being of survivors and family caregivers. However, few interventions are designed to strengthen the relationship between members of the care dyad in order to prevent depression and other poor outcomes. The aim of this study was to feasibility test a quality of relationship intervention for stroke dyads called Hand in Hand (HiH). Sixteen survivor-caregiver dyads were recruited and randomized into either the HiH intervention group (n=8) or the Information, Support, and Referral (ISR) control group (n=8). HiH dyads received up to 8 sessions with a social worker in-person, by telephone, or by Zoom web conference, prioritized according to a 17-item screening tool with 17 corresponding HiH content areas. ISR dyads received up to 8 sessions that included information, active listening, and referrals as needed. Process, satisfaction, and pre/post outcomes data were collected for both groups. Seventy-five percent of HiH dyads completed over half the sessions which lasted, on average, 55 minutes (range 26-76). Sixty-two percent of ISR dyads completed over half the sessions which lasted, on average, 21 minutes (range 15-33). Dyads in both groups reported being satisfied with the program materials and processes. Survivors and caregivers in both groups experienced improvements in outcomes, particularly caregivers in the HiH group who showed improvements in communication, coping, subjective relationship quality, and depressive symptoms. Findings suggest that HiH is feasible to implement with stroke dyads and has promise for improving outcomes for participants. Additional research is needed to determine program efficacy.

